# Physical activity to reduce cardiometabolic risk in veterans and their families: protocol for an interdisciplinary investigation with toolkit co-design

**DOI:** 10.3389/fspor.2025.1716823

**Published:** 2026-01-27

**Authors:** Laura J. Ferris, Philip Hawke, Emma Knight, Nicola W. Burton, Shelley E. Keating

**Affiliations:** 1 The University of Queensland, St Lucia, QLD, Australia; 2 The Australian National University, Canberra, ACT, Australia; 3 Griffith University School of Applied Psychology, Nathan, QLD, Australia; 4 Griffith Centre for Mental Health, Nathan, QLD, Australia

**Keywords:** cardio-metabolic, cardiovascular disease, exercise, ex-service, group norms, military, social support

## Abstract

Many veterans experience a decline in their physical activity following their separation from service. Contributory factors include changes in lifestyle, routines and occupational activities, or physical injury, disability, or mental ill health; whether service-related or otherwise. Low physical activity increases risk of serious health consequences, including cardiometabolic disease, which veterans experience at more than two-fold-higher rates than their general population counterparts. Psychological and social factors such as social identities, group norms and social support are important for physical activity uptake and maintenance, but are understudied in veteran populations and their families, especially alongside the incorporation of co-design principles and methods. This protocol paper presents a three-study research program aimed at improved management of cardiometabolic risk for veterans—with a focus on understanding barriers and enablers of physical activity, and culminating in the co-design of a physical activity toolkit. First, a cross-sectional survey will identify risk and protective factors associated with physical activity for veterans and their close family members, including psychosocial factors, such as norms about physical activity within veterans' families. Second, semi-structured interviews with at-risk veterans and family members will further examine perspectives and lived experience of barriers and enablers of physical activity uptake and maintenance. Third, focus group workshops will be undertaken to co-design a toolkit on physical activity that is evidence-based and acceptable for veterans with cardiometabolic risk and their families. Together, the research is expected to generate a new understanding of how best to support veterans and their families with uptake and maintenance of physical activity, particularly those with cardiometabolic risk indicators.

## Introduction

Cardiometabolic risk is a calculus of modifiable and non-modifiable risk factors, including perturbations in metabolic and cardiovascular function, which increase the likelihood of incident vascular events or the development of type 2 diabetes or cardiovascular disease. While evidence from non-English speaking and developing countries is scarce, research from “Five-Eyes” nations (i.e., US, UK, Australia, Canada and New Zealand) generally shows veterans are at greater risk of cardiometabolic disease and associated health outcomes than their population age-peers. For instance, US veterans are more likely than non-veterans to be living with obesity [43% vs. 34%, *p* < 0.01 (1)], which is a leading risk factor for the development of cardiovascular disease and metabolic disorders. Coronary heart disease is the most prevalent chronic disease in the Australian Defence Force (ADF), and one of the top three leading causes of death in each ADF service status group. When compared with the Australian general population, military service veterans are 2.5 times more likely to have a heart attack, stroke or vascular disease, and twice as likely to have type 2 diabetes (2).

### Exacerbating factors for cardiometabolic risk in veterans

There is evidence of disparities in the risk of cardiometabolic disease within veteran populations (3, 4). Comorbidities associated with cardiometabolic risk include physical and mental health concerns. For example, veterans with post-traumatic stress disorder experience high rates of obesity, dyslipidaemia, hypertension, type 2 diabetes, and cardiovascular disease (3). In a large cohort of veterans in the United States (*N* = 16,452), those who had traumatic brain injury reported higher rates of cardiometabolic health concerns, poorer physical functioning, and increased health care utilisation than those without (4).

The risk of cardiometabolic disease is likely different for serving-members vs. veterans due to the physical standards required for recruitment and active engagement in military settings (5). Active military service involves moderate to high levels of physical activity from training, operations, active duty and/or deployment (6). The transition from service to civilian life can involve substantial changes to lifestyle, occupational activities, social support and living conditions (7), including a decrease in physical activity levels (8).

### Physical activity in the management of cardiometabolic disease

Physical activity is an integral component of the prevention and management of cardiovascular and metabolic disease (9). Exercise is a planned and structured form of physical activity described as a “polypill” for its wide-ranging health benefits. Exercise produces clinically meaningful reductions in visceral adipose tissue and ectopic fat stores, which are hallmark pathophysiological drivers of cardiometabolic disease severity (10). Among other health benefits, regular exercise improves cardiorespiratory fitness and mental health (9). An increase in fitness by just a single unit (i.e., 1 mL/kg/min in maximal rate of oxygen consumption) is associated with a 10% reduction in cardiovascular mortality (11).

The benefits of exercise—and physical activity more broadly—have been observed in veteran populations. Within a small US veteran sample, higher cardiorespiratory fitness was associated with better cardiometabolic health (i.e., lower HbA1c, lower blood pressure, higher high-density lipoprotein cholesterol). Moreover, those with higher cardiorespiratory fitness had significantly lower post-traumatic stress disorder symptom severity compared with low and moderately fit veterans (12). Although cross-sectional, these findings provide physiological evidence on the importance of physical activity in a veteran sample. Veteran-relevant comorbidities such as post traumatic symptoms may present barriers to physical activity uptake and maintenance (13), but can also provide valuable indices of change on which physical activity improvements can be measured.

### Barriers and enablers of physical activity for veterans

Despite the benefits, many people do not take up physical activity at the recommended levels, i.e., equivalent of 150 min of moderate intensity physical activity with 2 days of muscle strengthening exercise per week. For instance, in Australia more than half of the general population is insufficiently physically active (14). Furthermore, to maintain the benefits of exercise, people need to sustain exercise. There are common general barriers to uptake and maintenance of physical activity, such as *lack of time*, *constrained access* to exercise services or other opportunities for physical activity, and features of the *physical environment*, such as poor urban design (15, 16). For people with cardiometabolic conditions, there are additional barriers to exercise, including comorbidities, musculoskeletal conditions, lack of exercise specialist support, and low exercise-related self-efficacy (17).

Veterans can encounter specific barriers to the uptake and maintenance of recommended levels of physical activity following their service. Veterans can be managing physical injury or disability from service (18); chronic pain (19); or may experience ongoing impacts of service-related psychological injuries and trauma affecting their social, emotional or occupational functioning (20). Service-related health issues and sequelae may emerge over time and not only at the point of exit from service. However, specific barriers and enablers of lifelong physical activity in veterans have not been well documented or theorized. In wounded, injured and/or sick military veterans from the UK, poor mental health, negative beliefs about physical activity, and low beliefs about physical capability were prominent barriers to physical activity (13). Low self-efficacy was reported as a key barrier in veterans with lower limb loss (18). Group exercise settings have triggered post-traumatic stress disorder which inhibited participation in lifestyle interventions despite an intrinsic drive to be healthy (21). In these circumstances, trauma-informed exercise approaches might be particularly important for enabling exercise uptake and enduring behavior change.

Social processes are also underutilized in efforts to improve physical activity uptake and maintenance (22). There is increasing acknowledgement of the role that veterans' social groups play in enhancing desired health behaviors and outcomes, not only during civilian transition but also in sustaining lifelong healthy practices (23). Peer-led interventions show promise (24) but remain under-researched. In particular, family members are a group that is underutilized in physical activity research and intervention, acknowledging that family members themselves can benefit from support to provide optimal care to their veteran loved ones (25).

Altogether, there is a need to address these research gaps and provide tailored, evidence-based tools to increase physical activity for at-risk veterans. Biopsychosocial or integrated interventions specifically addressing veterans' physical activity remain a neglected topic, and poor uptake and attrition can present an obstacle for veteran-focused interventions at large (26). A recent scoping review identified a lack of computer-based interventions that target veterans' physical health, with current interventions overwhelmingly focusing on mental health outcomes (27). Evidence-based and consumer-informed tools are needed so that at-risk veterans can enhance their uptake and maintenance of physical activity after service ends. These should be co-designed so they are acceptable and suitable for use by veterans, their families, and their supporting health professionals.

### The current research

Overall, there is a paucity of research examining the perceptions and experiences of physical activity and exercise care of veterans with, or at risk of, cardiometabolic disease. Given the prevalence of cardiovascular disease in this population, understanding the barriers and enablers for exercise adoption and maintenance for veterans is needed, and should inform the development of tailored resources to support lifelong exercise to help prevent and manage cardiometabolic disease in veteran populations. The role of family members in relation to veterans' physical activity uptake and maintenance also requires empirical investigation.

This protocol presents a proposal for a multi-method investigation of physical activity in veterans with cardiometabolic risk and their families. The research comprises two key phases: discovery and development. *Discovery* will involve qualitative (semi-structured interviews) and exploratory quantitative (survey) components to investigate barriers and enablers of physical activity, and to quantify putative risk and protective factors of physical activity. *Development* will engage veterans in focus group format to co-design a toolkit to facilitate and enhance physical activity uptake and maintenance, drawing from the insights obtained from these investigations.

## Method

### Design

This study will use a multi-method approach comprising qualitative and quantitative components supported by a generative co-design framework (28). First, a cross-sectional survey will be implemented to identify risk and protective factors associated with physical activity for veterans and their close family members. Second, semi-structured interviews will be carried out individually with at-risk veterans and family members to further elaborate perspectives and lived experience of barriers and enablers of physical activity.

Third, focus group workshops will be undertaken to co-design a toolkit on physical activity that is evidence-based, acceptable and satisfactory for veterans with cardiometabolic risk and their families. The workshops will employ co-design principles (29) to engage veterans in the co-production of the toolkit. In co-design, end-users are seen as equal partners, actively involved in the co-production of outputs, and their ideas are meaningfully integrated into the design process (30, 31). The workshops will employ the six co-design mindsets: (1) elevating lived experience, (2) practicing curiosity, (3) offering generous hospitality, (4) being in the grey (comfort with uncertainty), (5) learning through doing, and (6) valuing many perspectives (29).

**Figure 1:**
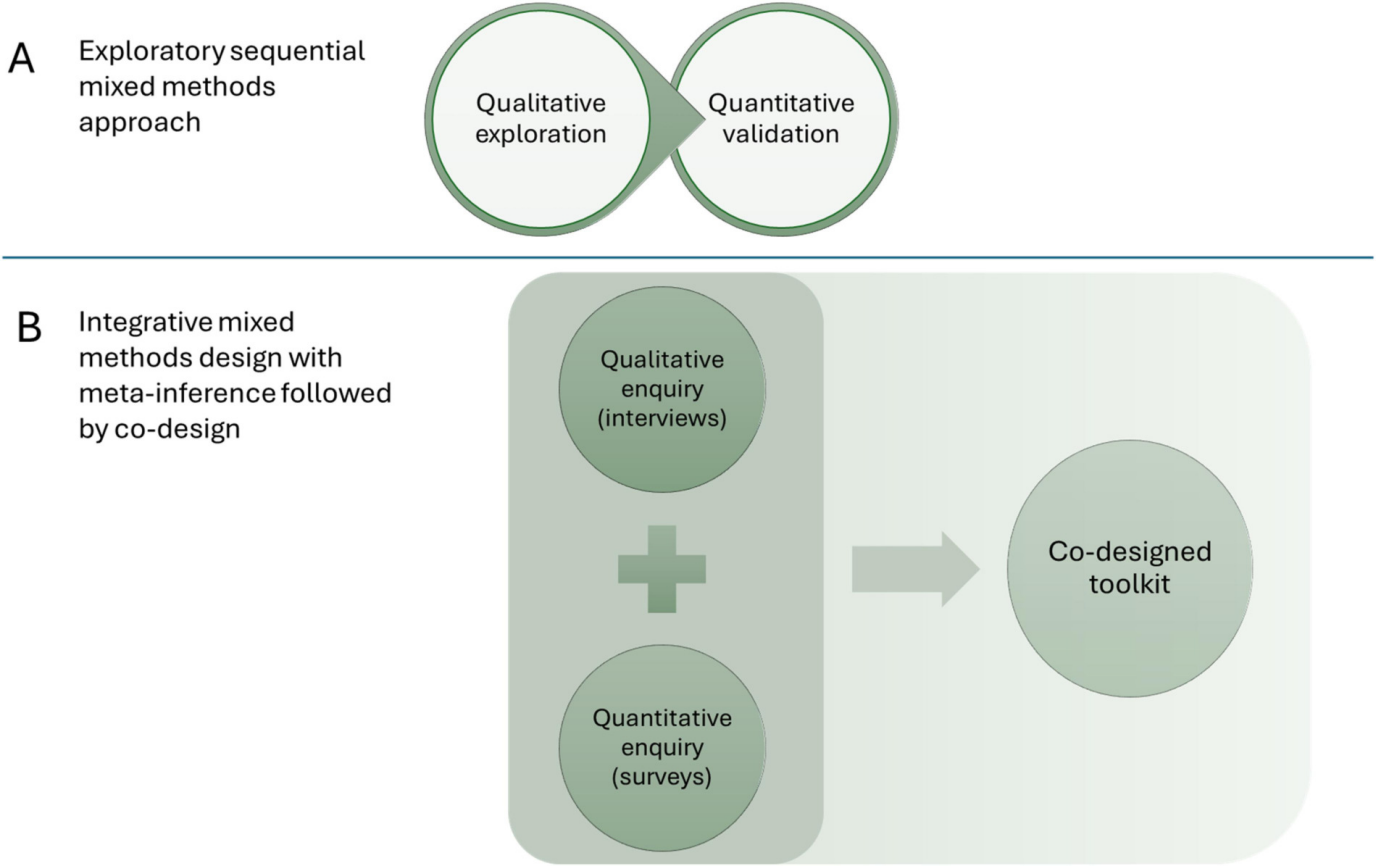
Visualisation of exploratory sequential mixed methods approach **(A)** (32); and integrative mixed method with meta-inference followed by co-design **(B)** (33). The current research proposal utilises an integrative mixed methods approach with co-design.

With this design, we adapt the traditional exploratory sequential mixed methods approach [i.e., qualitative exploration followed by quantitative validation (32); see Figure 1, Panel A] in favour of an integrative mixed methods approach that allows us to draw *meta-inferences* (33) before proceeding with co-design workshops (Figure 1, Panel B). Findings from the survey and semi-structured interviews will provide insights from veterans and families; these will be integrated to support the extraction of meta-inferences so that the combination of qualitative and quantitative insights add value beyond each component alone (33). For this reason, extensive elaboration on the co-design activities is not presented in the current protocol—because these will be informed by the findings of the earlier components. However, we describe process considerations and the analysis strategy further below (see Measures, Analysis strategy).

### Participants

Participants will be adult ex-service veterans aged over 18 years who are at risk of cardiometabolic disease and immediate adult family members (spouse or first-degree relative) of veterans. Cardiometabolic risk will be determined via satisfaction of one or both of the cardiometabolic risk criteria (self-reported waist circumference and physical activity level, see Table 1). Exclusion criteria are current service in any capacity in military or defence services, and not having access to relevant technology for online interviews, surveys, or focus groups. Participants may be both veterans and a family member of a veteran, in which case they may choose in which capacity they wish to take part. All participants will receive information on support services should they be required.

**Table 1:** Cardiometabolic risk criteria.

Variable	Criteria for elevated risk
Waist Circumference	Men:
	≥94 cm (increased risk) ≥102 cm (substantially increased risk)
	Women:
	≥80 cm (increased risk) ≥88 cm (substantially increased risk)
Physical Activity	<150 min of moderate intensity physical activity per week

In the Discovery phase (survey, interviews), participants will be veterans with cardio-metabolic risk factors and veterans' adult family members. To ensure inclusiveness, there is no requirement for participants to present in dyads; individual veterans and family members will also be able to participate. The online survey will aim for a target *n* = 80. This target sample size is based on a sensitivity power analysis using G*Power (34). A sample of *n* = 68 should provide sufficient power at 0.8 to detect to detect cross-sectional associations with physical activity measures (*f*^2^ = 0.15) using multivariate linear regression with two predictors and *α* = .05. One-on-one semi-structured interviews will aim for a target *n* = 15–30, contingent on thematic saturation). In the Development phase, the participants will be veterans with cardiometabolic risk factors. Focus groups (target *k* *=* 2, *n* = 12) will be conducted virtually.

### Measures

#### Survey

An online survey will collect data on self-reported waist circumference, weight and height; perceived cardiometabolic risk; physical activity and physical activity preferences; mental health factors (psychological distress, wellbeing, depression, anxiety, and post-traumatic stress), social factors (physical activity social norms, social identification, and social support); and demographics (including age, gender, employment status, service duration, rank on exit).

**Waist circumference.** Participants will be invited to assess and self-report their waist circumference in centimetres using a standard gender-informed waist measurement protocol (measured midway between the lower rib margin and iliac crest to the nearest ½ centimetre). Participants will also self-report their weight and height. Cardiometabolic risk will be derived from meeting one or more of the criteria listed in Table 1. In case of missing waist circumference data, body mass index (BMI, kg.m^−2^) will be calculated and classified as increased risk if BMI ≥30 kg.m^−2^; however, BMI will not be used as a primary criteria due to its crude ability to inform cardiometabolic disease risk at an individual level.

**Physical activity** will be assessed using the Active Australia Survey (AAS), a validated tool developed by the Australian Institute of Health and Welfare (AIHW). The AAS is a self-report measure of frequency and duration of physical activity in the past week, including walking, moderate, and vigorous activities. The AAS shows acceptable reliability and validity in assessing physical activity levels in the general population (35).

**Physical activity context preferences** will be assessed using items used by Burton, Khan (36). Preferences in various elements of physical activity, such as format, location, and social setting, will be measured with 23 items on a 5-point Likert scale (Strongly disagree to Strongly agree); for example, “I prefer physical activities that are done on my own,” “are competitive,” and “include a social aspect”.

**Psychological distress** will be measured using the Kessler Psychological Distress Scale (K10). This is a 10-item questionnaire assessing frequency of anxiety and depressive symptoms experienced by respondents in the past 30 days. Each item is rated on a 5-point scale ranging from “None of the time” to “All of the time”. Higher scores indicate greater psychological distress. The K-10 is widely used in both clinical and research settings and shows excellent psychometric properties in diverse populations (37).

**Well-being** will be assessed using the Mental Health Continuum-Short Form (MHC-SF). This is a 14-item measure of emotional, psychological, and social well-being. Respondents rate the frequency of their experiences of positive feelings and functioning over the past month on a 6-point scale ranging from “Never” to “Every day”. The MHC-SF has been validated across various populations and is a reliable measure of positive mental health (38). Higher scores indicate greater wellbeing.

**Post-Traumatic Stress** symptoms will be measured using the Short Post-Traumatic Stress Disorder Rating Interview (SPRINT). The SPRINT is an 8-item self-report scale assessing post-traumatic stress symptoms, including re-experiencing, avoidance, hyperarousal, and functional impairment. Each item is rated on a 5-point scale from “Not at all” to “Very much” (39).

**Social group norms.** A bespoke measure will be used to assess shared social norms on physical activity within close social groups or family. The measure comprises four items measuring the degree to which physical activity is perceived as important, central, and regularly practiced within the group. Respondents will be asked to reflect on their immediate family/partner or other close contact and indicate their level of agreement with the following statements on a 7-point Likert scale (“1—Not at all” to “7—Very much so”):

“Physical activity is important to us.”
“We make sure to get enough physical activity each week.”
“Physical activity is a big part of our lifestyle.”
“In my family, physical activity is central to who we are.”

**Social identification and social support.** Identification with relevant social groups will be assessed using the single-item measure of social identification (40), adapted for each group (including Defence community, veterans, workplace, local neighbourhood). Additional items will assess perceived social support from these groups. Responses will be on a 7-point scale ranging from “1—Not at all” to “7—Very much so”; for example, “I identify with the Defence community”, “I feel supported by veterans”, “I identify with my local neighbourhood”.

#### Interviews

Veteran and family member participants will be individually interviewed in a semi-structured format in accordance with the interview protocol (Supplementary Materials). Consenting participants will be asked to provide brief socio-demographic information, then complete one-on-one interviews with a researcher-interviewer. Participants are provided with Interviews will be recorded, transcribed and identifying information removed. Interview data will undergo inductive thematic analysis.

#### Co-design

Co-design workshops will focus on developing a shared understanding of exercise-related issues and solutions to enable physical activity among veterans and their families. Consenting participants will be asked to provide brief socio-demographic information. Co-design materials, activities and runsheet will be developed in response to the pending results and findings of the discovery phase (surveys, interviews).

In the development and co-design phase, the findings from the discovery phase will be integrated with the current evidence-base on barriers and enablers of physical activity uptake and maintenance in veteran populations. In preparation, the research team will develop community-facing knowledge exchange materials (summaries, activities, discussion points) and an instructional participant guide for virtual focus groups; and test virtual systems with participants to ensure connectivity and functionality. In the co-design sessions, a trained co-design facilitator from the research team will engage with veterans in focus group format to iteratively co-design resources to support physical activity uptake and maintenance for veterans with high cardio-metabolic risk and their families.

### Procedure

Veterans and their family members will be recruited via veteran networks, social groups and communities including via The Gallipoli Medical Research Foundation and its associated veteran and family networks, relevant Facebook or other social media groups, and other professional contacts. Participants will be invited to share study information to their veteran networks. A study website will provide a method for individuals to contact the research team directly to become involved. All communication will emphasise that participation is voluntary and anonymous. Prospective participants will be invited to review the study website and the Participant Information and Consent Form to decide whether to participate.

Online surveys will be delivered via Qualtrics. Interviews will be conducted virtually via Zoom or Teams and last 30–60 min. All interviews will be recorded and transcribed prior to thematic analysis. Focus groups will be conducted virtually, with the runsheet to be developed based on findings from the Discovery phase.

Focus groups will be recorded and transcribed, and group artefacts (typed comments and notes) will be collected and integrated into the analyses.

### Analysis strategy

In the discovery phase, **qualitative data** will be subjected to thematic analysis following Braun and Clarke's (41) inductive approach, including familiarisation, coding, theme development, and refinement. A subset of the data will be double-coded by another team member and any discrepancies addressed, and themes will be subjected to consensus-based discussion by the multidisciplinary research team. Lived experience feedback on themes (member checking) will be invited but not mandated. For **quantitative survey data,** descriptive statistics will be used to characterise the sample and multiple regression analyses to investigate cross-sectional associations between cardiometabolic risk (waist circumference, physical activity) and the demographic, mental health, social and other variables. Exploratory moderation analyses will be conducted to identify boundary conditions.

In the **integration phase**, the insights from the qualitative and quantitative studies will be summarised and cross-validated to identify convergence and divergence. Meta-inferences will be gleaned through team examination, discussion and reflection upon the integration material. As noted above, the findings from the discovery phase will be further integrated with the current evidence-base on barriers and enablers of physical activity uptake and maintenance in veteran populations.

Finally, in the **codesign phase**, community-facing knowledge exchange materials will be developed to facilitate *discussion*, *knowledge exchange* and *co-creation* at the co-design workshops. This aspect of the workshop proceedings is intended to be informed by the co-design principles, in particular, elevating lived experience; practising curiosity; and being in the grey; and valuing many perspectives (29). The co-design outputs are intended to be collaborative, responsive and driven by the perspectives, needs and preferences of veterans. To that end, focus group transcripts and artefacts will be collected, summarised and thematically analysed to produce co-designed recommendations and resources (the *toolkit*).

## Ethical considerations

The study received ethical approval from the Department of Defence and Veterans Affairs Human Research Ethics Committee (HREC) on 8 October 2024 (BN78496952, #590-24) and subsequently ratified by the University of Queensland's HREC. Principal ethical considerations relate to ensuring that participants are supported to provide their voluntary informed consent, and that participant data is appropriately handled to protect privacy and preserve anonymity. The study will seek voluntary informed consent from participants through an online consent form. Participants will be assured they can withdraw at any stage, with data deleted upon request unless already anonymised, and that their participation or otherwise will be confidential. Participants will receive information on support services should they be required. All data will be stored securely on the University's Research Data Manager (RDM) system, accessible only to the approved research team. Only de-identified data will be published, and data will be pooled and reported in aggregate where small subsamples create any risk of re-identification and any individual quotes anonymised. Although participants are not expected to experience any elevated risk or any harm arising from their participation in the study, participants will be offered support contacts in the event they experience any disturbance to their wellbeing or discomfort after taking part. Remuneration will be in the form of a $20 voucher for survey participation, and $50 voucher for interview or focus group participation; with a view to acknowledging participants' time and their contributions to new knowledge as part of the study, guided by best practice consumer participation remuneration policies (42).

## Expected outcomes and dissemination

The key outcome of the research is to support the development of a bespoke toolkit to support physical activity for veterans with cardiometabolic risk and their families. The research will be communicated to veteran networks, organisations, families, communities, and participants themselves who may benefit from new insights on physical activity uptake and maintenance derived from the research. The research process and findings will be disseminated via peer-reviewed publications; conference presentations; practitioner, industry and consumer briefings; and other community outreach where relevant and feasible. The study is based in Australia and is expected to capture both region-specific and generalisable insights. Future research may also replicate and extend the current proposal in other regions and populations.
